# Applying Next-Generation Sequencing and Multi-Omics in Chronic Obstructive Pulmonary Disease

**DOI:** 10.3390/ijms24032955

**Published:** 2023-02-03

**Authors:** Pei Yee Tiew, Oliver W. Meldrum, Sanjay H. Chotirmall

**Affiliations:** 1 Department of Respiratory and Critical Care Medicine, Singapore General Hospital, Singapore 169608, Singapore; 2 Duke-NUS Graduate Medical School, Singapore 169857, Singapore; 3 Lee Kong Chian School of Medicine, Nanyang Technological University, Clinical Sciences Building, 11 Mandalay Road, Singapore 308232, Singapore; 4 Department of Respiratory and Critical Care Medicine, Tan Tock Seng Hospital, Singapore 308433, Singapore

**Keywords:** COPD, microbiome, next generation sequencing, multi-omics, 16S, ITS, metagenomics

## Abstract

Microbiomics have significantly advanced over the last decade, driven by the widespread availability of next-generation sequencing (NGS) and multi-omic technologies. Integration of NGS and multi-omic datasets allow for a holistic assessment of endophenotypes across a range of chronic respiratory disease states, including chronic obstructive pulmonary disease (COPD). Valuable insight has been attained into the nature, function, and significance of microbial communities in disease onset, progression, prognosis, and response to treatment in COPD. Moving beyond single-biome assessment, there now exists a growing literature on functional assessment and host–microbe interaction and, in particular, their contribution to disease progression, severity, and outcome. Identifying specific microbes and/or metabolic signatures associated with COPD can open novel avenues for therapeutic intervention and prognosis-related biomarkers. Despite the promise and potential of these approaches, the large amount of data generated by such technologies can be challenging to analyze and interpret, and currently, there remains a lack of standardized methods to address this. This review outlines the current use and proposes future avenues for the application of NGS and multi-omic technologies in the endophenotyping, prognostication, and treatment of COPD.

## 1. Introduction

**Figure 1:**
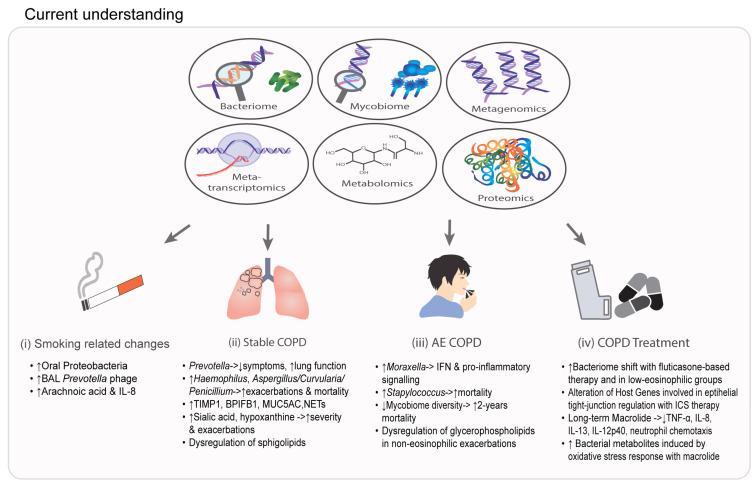
A pictorial summary of the current literature in relation to the application of next-generation sequencing (NGS) and multi-omic analysis in (i) smoking-related change [12,13,14], (ii) stable COPD [15,16,17,18,19,20,21,22], (iii) acute exacerbations of COPD (AE COPD) [16,23,24,25], and (iv) COPD treatment [26,27,28]. BAL: bronchoalveolar lavage, IL: interleukin, TIMP1: metalloproteinase-inhibitor 1, BPIFB1: bactericidal/permeability-increasing fold-containing family B member 1, MUC5AC: mucin 5AC, NETs: neutrophil extracellular traps, IFN: interferon, TNF: tumor necrosis factor, ↑: increase, ↓: decrease.

The application of next-generation sequencing (NGS) has improved our understanding of the respiratory microbial ecosystem in health and disease [1,2]. Traditionally, the identification of microorganisms has been achieved through culture, which often requires a substantial microbial load for reliable detection. Since NGS was first employed to evaluate respiratory specimens in the early 2000s, researchers have gained a deep understanding of the potential role of the lung microbiome in chronic obstructive pulmonary disease (COPD) [2,3,4]. The dynamic nature of the lung microbiome, influenced by environmental and host factors, treatment, and disease status, has led to the use of multi-omics approaches to better delineate the complex microbial communities that exist in COPD [1]. Over recent times, microbiome research has evolved into exploring multi-kingdom and host–microbial interactions using a combination of NGS and multi-omics. One commonly used technique to assess microbial communities in the airway is targeted amplicon sequencing, an approach that amplifies specific regions of bacterial (16S ribosomal ribonucleic acid (rRNA)) or fungal (18S internal transcribed spacer (ITS)) DNA using specific primers followed by sequencing and taxonomic classification [5,6]. Alternatively, shotgun metagenomic sequencing performs an unbiased sequencing of all DNA in a particular sample, providing a more holistic and balanced view while offering additional functional and/or resistance information about the detected microbes. Metatranscriptomics involves RNA sequencing that provides information about microbial viability and gene expression in addition to potential host–microbe interaction when host RNA is incorporated [7]. To gain a comprehensive understanding of inflammation and cellular pathways involved in COPD pathogenesis and progression, researchers may also employ genomic, proteomic, lipidomic, and metabolomic assessments, each representing different views of a single clinical case [8,9,10]. While each individual omic approach has value, a systems medicine perspective combining several multi-omics may provide even greater insight, especially when appropriate integration for clinical outcome prediction is used [11]. Importantly, the high inherent dimensionality and data heterogeneity between the various omics pose analytical and interpretation challenges that require the development of appropriate strategies for data integration and analysis. Here, we provide a review of the current and potential future use of NGS, multi-omics, and their data integration across the different stages of COPD (Figure 1).

## 2. NGS in Endophenotyping and Prognostication of COPD

Smoking-related change. The oral bacteriome in the healthy individual is dominated by the genera *Streptococcus*, *Prevotella*, and *Veillonella* [12,13]. Studies have reported that smoking is associated with changes in the oral microbiome, including a decreased abundance of the Proteobacteria phylum and the genera *Neisseria, Porphyromonas, Gamella, Capnocytophaga, Peptostreptococcus,* and *Leptotrichia* compared to healthy non-smokers [12,13,29,30,31]. In addition, functional pathways related to carbohydrates, energy, and xenobiotic metabolism are depleted in smokers [13,29]. Interestingly, the oral bacteriome appears to be similar between former smokers and never smokers [29]. Such change has not been observed in the lower respiratory tract where bacterial microbiome profiles are similar between smokers and non-smokers [32,33,34]. These data suggest that changes to the oral bacteriome are not permanent and are potentially reversible with smoking cessation and likely do not involve the lower airway before a COPD diagnosis. In a small study of the virome, Proteobacteria and Firmicute bacteriophages represent the main DNA viruses present in the airway, and their abundance is increased in smokers, correlating with increased levels of interleukin-8 (IL-8) and arachidonic acid [14]. This implies a potential role for bacteriophages in the induction of inflammation in the airways of active smokers. Limitations of virome studies include their low abundance in the airway, incomplete virome databases, and the lack of standardized methodology for virus detection. These have all hindered the progress and understanding of its contribution to chronic respiratory diseases [35]. Further research is, however, needed to determine the precise relationship between these microbial and inflammatory changes and the development of COPD.

Mild–moderate COPD. While the lower airway bacteriome remains comparable between non-COPD smokers and healthy individuals, changes are observed in patients with COPD relative to non-COPD smokers [35]. Studies report a decreased bacterial alpha diversity (within sample diversity) in individuals with COPD, and this diversity is further altered during acute exacerbations (AE COPD). Analysis of bronchoalveolar lavage (BAL) and bronchial brushing samples reveal an increased abundance of *Streptococcus*, *Lactobacillales, Fusobacterium,* and *Moraxella* in mild to moderate COPD and *Prevotella* in those without COPD [15,36,37]. *Prevotella* is therefore considered a “healthy” microbe, and its decreased abundance in COPD is linked to increased disease severity and a downregulation of genes promoting host defense as observed through transcriptomic analysis [15]. *Madapoosi* and colleagues integrated BAL bacteriomes and metabolomic data from the SPIROMICS cohort in individuals with mild–moderate COPD and showed that the occurrence of *Prevotella* and its associated compounds, adenosine and 5- methylthioadenosine (MTA), predict clinical outcome in COPD with lower symptom scores and higher lung function. Adenosine regulates airway surface liquid (ASL) and plays a key role in maintaining ciliary function and mucus clearance, while MTA is involved in purine metabolism and methionine salvage [38]. In contrast, the presence of *Streptococcus, Neisseria,* and *Veillonella*, along with their associated metabolites in the classes of glycosphingolipids, glycerophospholipids, polyamines, and purine metabolism, were correlated with poorer lung function and higher COPD symptoms, suggesting that altered host–microbe interactions are observed even at the earliest stages of this disease [37].

Moderate–severe COPD. The dominant phyla in moderate–severe COPD include Proteobacteria and Firmicutes and the genera *Haemophilus*, *Moraxella, Streptococcus, Rothia, and Staphylococcus* [39,40,41]. As COPD progresses, further decreases in bacterial alpha diversity are observed, most prominently in severe COPD [39,40]. Increased *Pseudomonas* is also observed in COPD with severe airflow limitation [42]. In addition, microbial variation is associated with airway inflammation and disease severity [23,39,41,43,44]. Airway *Haemophilus* dominance is linked to neutrophilic inflammation, the activation of neutrophil extracellular trap (NET) formation, and the induction of interleukin-6 (IL-6) signaling [45,46]. Studies integrating microbiome and proteomic data illustrate that the upregulation of proteins involved in neutrophil activation pathways is most common with Proteobacteria-dominant microbiomes, which in turn associate with an increased exacerbation frequency, disease severity, and overall poorer COPD prognosis [23]. Patients with frequent COPD exacerbations tend to have elevated levels of microbial genes involved in lipopolysaccharide biosynthesis and energy metabolism [47,48,49]. When the airway mycobiome (fungal microbiome) is considered, a high abundance of *Candida* and distinct fungal profiles are observed in COPD, with *Aspergillus*, *Curvularia,* and *Penicillium* having association with poor clinical outcomes, including exacerbations and even mortality [16,24,50]. *Candida* is the dominant genera in the COPD airway with an increased abundance observed in older individuals and COPD compared to healthy controls [24,51,52]. Colonization with *Candida* is associated with lung function decline and exacerbations in cystic fibrosis, but their roles have been lesser explored in COPD [53]. Taken together, these findings support the presence of microbiome signatures and the important individual roles of bacteria, fungi, metabolites, and host interaction in the development, progression, prognosis, and exacerbations associated with COPD.

COPD exacerbations. During AECOPD, the mycobiome remains relatively stable; however, bacteriome composition may be altered, often influenced by underlying airway inflammation and baseline microbial profiles. Such changes to the bacteriome may have significant consequences on the clinical severity and/or outcomes of exacerbations. A large, multi-center, longitudinal study combining data from the BEAT, COPDMAP, and AERIS cohorts identified two neutrophilic bacteriome endotypes in individuals with COPD [54]. The first, dominated by *Haemophilus*, associates with high levels of sputum interleukin (IL)-1β and tumor necrosis factor (TNF)-α and remains stable during exacerbations [54]. The second endotype, characterized by a balanced bacteriome and increased levels of IL-17 in the sputum and blood, is more prone to bacterial shifts during exacerbations, either towards the neutrophilic-*Haemophilus* endotype or a more eosinophilic driven endotype where *Campylobacter* and *Granulicatella* are favored [54]. The dynamic bacterial changes therefore observed with exacerbation, at least in a subset of patients with COPD, may therefore be determined by their underlying inflammatory endotypes; however, where specific genera of bacteria are evaluated, *Moraxella* has been associated with AECOPD and upregulation of interferon and pro-inflammatory signaling as observed in longitudinal studies using transcriptomics and proteomics analysis [55]. Interestingly, profiles with elevated *Moraxella* reverse in the post-exacerbation state [55]. In severe exacerbations, elevated *Staphylococcus* relates to increased mortality, while *Staphylococcus* and *Pseudomonas* associate with exacerbations and greater disease severity [41,42,43]. By contrast, *Veillonella* is reduced during AECOPD and relates to better survival [17,41,56]. While little alteration is observed in the airway mycobiome profiles between stable, exacerbation, and post-exacerbation COPD states, a reduced alpha diversity in the COPD mycobiome detected during exacerbations does associate with an increased two-year mortality, suggesting that airway fungal change remains an important consideration [16,18].

## 3. The Application of Proteomics and Metabolomics in COPD

Cigarette smoking has direct effects on the airway protein and/or metabolites that have implications for the development of COPD in smokers. Proteomic analysis of BAL fluid from smokers reveals alterations in over 500 proteins involving 15 molecular pathways compared to non-smokers [19]. In smokers, proteins involved in oxidative phosphorylation and the citrate cycle were upregulated, while ribosomal and antigen presentation-related proteins were downregulated. Phagosomal and leucocyte transendothelial migration (LTM) pathways correlate with airway obstruction, and CD8 CD69 T cells and their related proteins are dysregulated in smokers, while CD8 T cells associate with increased airway inflammation and remain critical in the development of emphysema [19,57].

Using induced sputum in four different groups (i.e., smokers, former smokers, never-smokers, and those with COPD), a gradient trend of altered airway proteins is observed, with the greatest change detectable in current smokers and individuals with COPD [58]. Smokers demonstrate an upregulation of mucin/trefoil proteins, xenobiotic metabolism enzymes, peptidase regulators, and proteins involved in the redox process, with such change decreasing in former smokers to the point of approaching levels of never-smokers [58]. Thirteen COPD-specific proteins can discriminate current smokers, including metalloproteinase-inhibitor 1 (TIMP1), bactericidal/permeability-increasing fold-containing family B member 1 (BPIFB1), and apolipoprotein A-I (APOA1) [58]. BPIFB1 protein, found in goblet cells, possesses antimicrobial and immune-related functions and is upregulated in the sputum of smokers with COPD and correlates with pack-years smoking exposure and declining lung function over a four-year period [20,59]. Mucus hypersecretion, a common (chronic bronchitis) phenotype in smokers and individuals with COPD, is associated with increased sputum MUC5AC in smokers and individuals with chronic bronchitis, while emphysema is characterized by high plasma protein levels and components of neutrophil extracellular traps (NETs) in sputum. Importantly, these changes are also observed in e-cigarette users and support the key roles of mucins and their related proteins in COPD pathogenesis and progression [57,60,61].

A multi-center sputum metabolomic study involving 980 patients, including smokers, non-smokers, and individuals with COPD, identified elevated levels of sialic acid, hypoxanthine, xanthine, methylthioadenosine, adenine, and glutathione analytes in COPD. These metabolites associate with mucus hydration, adenosine metabolism, and oxidative stress, while sialic acid and hypoxanthine are linked to disease severity, time to next exacerbation, and the prediction of future COPD exacerbations [21]. Paired BAL and plasma metabolomics in 115 individuals from the SPIROMICS cohort demonstrate that the two compartments have poor correlation, with BAL being more closely associated with COPD phenotypes. Multiple classes of metabolomes, including amino acids, fatty acid, carnitines, and phospholipids, associate with lung function and emphysema. Amino acids such as leucine and lysine are enriched in emphysema, while decreased levels of isoleucine, serine, and arginine associate with poorer lung function (FEV_1_/FVC ratio) [22]. Distinct metabolomic signatures are also characteristic of AECOPD, with reduced levels of tryptophan and increased activity of the Indoleamine 2,3-dioxygenase pathway, which possesses antimicrobial properties [25,62]. Altered airway metabolomics are observed even in stable COPD, with variation dependent on underlying COPD phenotypes and further change occurring during AECOPD.

Sphingolipids, dysregulated in COPD, are essential components of the plasma cell membrane and remain involved in cell homeostasis and the binding of microbial toxins [63]. Increased ceramide levels are found in the lung tissue of smokers and individuals with mild COPD; however, as COPD progresses to a more severe disease, reverse trends emerge [64]. Dysregulation of 13 sphingolipids observed in induced COPD sputum positively correlates with smoking pack-year exposure and reduced lung function [65]. These include ceramides, dihydroceramides, sphingomyelin, and glycosphingolipids, and these changes linked to smoking attenuate after two months of smoking cessation [65]. Changes in glycerophospholipids are observed during AECOPD, particularly in non-eosinophilic phenotypes [66]. Similar results are reported in plasma and serum metabolomics and proteomic studies, with the dysregulation of amino acid, phospholipids, and sphingolipid pathways in COPD and/or emphysema relative to smokers, which associate with declining lung function and increased exacerbations [26,27,67,68,69,70,71,72]. Proteomic and metabolomics signatures identified in COPD clearly differentiate from smokers without COPD, and several of these relate to the oxidative stress response, mucin production, lipid metabolism, leucocyte, and phagosomal pathways. All such pathways are relevant in COPD pathogenesis and represent potential targets for drug development.

## 4. Understanding COPD Therapeutics Using NGS

*Inhaled corticosteroids (ICS).* ICS therapy is recommended as add-on therapy for COPD patients experiencing frequent exacerbations and/or with a raised blood eosinophil profile; however, it is proposed to associate with risks of developing pneumonia. Changes in the lung bacteriome are linked to ICS use which may contribute to increased susceptibility to pneumonia. A randomized controlled trial (RCT) in steroid naïve COPD patients found that treatment with fluticasone-containing inhalers for one year was associated with increased alpha diversity and the presence of *Streptococcus pneumoniae* and *Haemophilus influenzae* but only in patients with low baseline sputum and/or blood eosinophil count [73]. The effects of ICS on *Streptococcus* abundance are thought to be mediated by cathepsin D/cathelicidin activity, which has roles in host defenses against bacteria in an ICS-treated mouse model [74]. In addition, changes to the lower airway bacteriome with increased growth of *Klebsiella pneumoniae*, *Pseudomonas aeruginosa, Stenotrophomonas maltophilia,* and *Acinetobacter baumanii* are observed with fluticasone-based therapy [75]. Fluticasone alters *Klebsiella pneumoniae* gene expression, leading to less virulence and evasion of the host’s innate immune response, leading to better airway survival [76]. Another RCT found that fluticasone-based therapy after 12 weeks was associated with greater bacterial shifts from baseline and decreased alpha diversity compared to the formoterol-only group [77]. Host transcriptomic–microbiomic analysis using the same cohort further found that fluticasone-based therapies associate with the enrichment of gene expression and accompanying pathways that relate to longitudinal innate and adaptive immune-inflammatory change and also to bacteriome diversity, in particular, *Haemophilus* in the fluticasone- but not budesonide- and/or formoterol-based groups [78]. Such change is thought to be steroid-specific, and alterations to lung microbiomes and host transcriptomes may play important roles in the occurrence of pneumonia following ICS administration in COPD [78]. A large cross-sectional study of mild–moderate COPD found that ICS therapy reduced the abundance of *Prevotella* and host genes involved in tight junction regulation using bronchial brushings; however, no differences in bacterial load and/or alpha diversity between ICS and non-ICS groups were observed [15]. Reported differences in the effects of ICS on the microbiome are likely influenced by sample type and method of collection (e.g., sputum versus bronchial brushings), COPD severity, dose and type of ICS used, and duration of treatment. Nonetheless, alterations in bacteriomes have been described in all studies, albeit with different magnitudes and specific bacterial species. Moreover, changes in the lung microbiome further influence the host transcriptome, supporting the alteration of gene expression secondary to microbial dysbiosis related to ICS use in COPD. Overall, these data collectively suggest that ICS, particularly fluticasone therapy, modulates lung microbiomes and in turn host transcriptomes. Interestingly, similar alterations have not been observed in lung mycobiome profiles with ICS use in COPD whether sputum or BAL was employed [16,24,79].

*Long term macrolides.* Macrolides are used in moderate–severe COPD with frequent exacerbation [28]. These drugs have anti-inflammatory, immunomodulatory, and antimicrobial effects; however, their long-term use increases the risk of developing antimicrobial resistance where studies have found macrolide resistance genes in COPD, particularly in eosinophilic predominant individuals [80,81,82]. *Streptococcus*, *Actinomyces*, *Campylobacter,* and *Aggregatibacter* are among the key microorganisms contributing to macrolide resistance [80,81]. Chronic antibiotic use in COPD associates with a greater than three-fold increase in antibiotic resistance, although, importantly, the overall bacterial burden is stable [83]. In an RCT where COPD patients were given either azithromycin or placebo, and BAL samples were collected at baseline and 8 weeks post-treatment, analyzed using 16S rRNA targeted amplicon sequencing, metabolomic and cytokines analyses revealed no significant differences in bacterial load between and within groups at baseline and 8 weeks. Importantly, however, the azithromycin group demonstrated a decreased alpha diversity and 11 low-abundance taxa, accompanied by increases in particular bacterial metabolites, including benzoic acid, indole-3-acetate, and glycolic acid. These changes, likely due to an oxidative stress response were accompanied by a decreased host inflammatory response, including chemokine ligand 1, TNF-α, IL-13, and IL-12p40, suggesting that azithromycin alters host–microbial interaction that subsequently leads to changes in bacterial metabolites, resulting in an overall anti-inflammatory effect [84].

*Short-term antibiotics and systemic corticosteroids.* During AE COPD, corticosteroids and antibiotics are often prescribed only for short durations, typically ranging from 5 to 7 days. Alteration in bacteriome profiles was observed with decreased bacterial diversity following oral corticosteroid treatment. This includes increases in the phyla *Proteobacteria* and genera *Haemophilus* and *Moraxella* but decreases in *Streptococcus* [44]. Reverse trends are observed with antibiotic treatment [44,85]. A decreased bacterial diversity with increased prevalence of *Pseudomonas* and *Stenotrophomonas* coupled with upregulation of microbial gene expression in xenobiotic metabolism and antimicrobial resistance are seen in groups of patients that fail to respond to antibiotics, defined as persistent or worsening signs and symptoms after 72 h of treatment during an AECOPD. In contrast, *Prevotella*, *Peptostreptococcus, Selenomonas*, and the gene responsible for DNA repair and amino acid metabolism are decreased in antibiotic failure groups [86]. Despite this, changes to the mycobiome are not observed following either antibiotic and/or corticosteroid therapy during an AECOPD, suggesting that short courses of systemic antibiotics and/or corticosteroids influence bacteria but not fungi in the COPD airway [16]. In light of this, it may be more optimal to consider interactions between different microbial kingdoms within a single organ compartment rather than simply evaluating a single kingdom. Such an approach may provide a better understanding of the complex microbial communities and their inter-relations. *Mac Aogain* and colleagues demonstrate altered inter-kingdom interactions following exacerbations and antibiotic treatment in bronchiectasis [87]. Future research using similar concepts and approaches should be extended to COPD where patient stratification and treatment effectiveness can be optimized for AE COPD using a combination of NGS and multi-omics [87].

## 5. Multi-Omic Data Integration in COPD

Omics technologies, including NGS and mass spectrometry, have made it possible to assess multiple aspects of a single patient’s biology, including their genome, transcriptome, epigenome, proteome, metabolome, and microbiome [10,37,88]. While analyzing one aspect in isolation can provide valuable information (Figure 1), it may offer an incomplete picture of the pathobiological mechanisms (endotypes) at play and their response to treatment, particularly during exacerbation and/or disease progression [44,51,89,90]. For example, COPD disease progression due to alpha-1 antitrypsin (AAT) deficiency is highly variable, and metabolites show poor statistical power for prediction after correction for false discovery rate [71,91]. Integrated approaches combining omics data in a sequential and/or simultaneous manner may alternatively provide a better, more holistic view of disease pathophysiology and allow for the search for novel biomarkers associated with particular endophenotypes. Considering multiple “omics” may allow a better understanding and/or prediction of COPD outcomes where the detection of analytes in one dataset may be enhanced by “borrowing” information from another. This leads to the identification of “treatable traits” and may allow targeted treatment in individual patients based on the understanding of their underlying molecular and microbial endophenotypes along with relevant clinical information and outcomes [92,93,94].

*Multi-omic integration strategies:* Several recent studies have used multiple omics datasets and other biological and/or clinical information holistically, analyzing them together using integrative methods to identify groups of patients with co-alteration of microbial and/or multi-omic profiles [37,92,95]. The hypothesis-generating power of multi-omics may be applied to inform in vivo experimental systems and uncover the role of particular lung microbiome-derived metabolites. For instance, changes in the tryptophan metabolism of *Lactobacilli* reduced indole-3-acetic acid, which in turn alleviated COPD-associated airway inflammation and epithelial apoptosis [95]. The intergration of culturomic strategies with metagenomics allows for mechanistic studies and a deeper understanding of microbial virulence and the mechanisms of antimicrobial resistance [96]. However, no single integration strategy is suitable for all purposes, and this has led to the development of various approaches, including multi-staged analysis and meta-dimensional analysis [97,98]. Multi-staged integration involves considering two numerical and/or categorical features of the data, while meta-dimensional analysis incorporates all relevant data types by combining them into a single matrix or “metadata” set that can be holistically assessed. While meta-dimensional analysis possesses greater statistical power, it may be more difficult to implement when combining data from different types of datasets [87,99]. This can be achieved using a similarity network fusion (SNF) approach, which involves the creation of similarity networks between different omics datasets followed by fusion and analysis of the integrated networks as successfully applied in bronchiectasis and COPD [11].

*Technical and computational challenges*: Technical limitations in clinical research and the accompanying data may lead to the analysis of only a fraction of the biological variability present between individuals. In all intergrative analyses, unaccounted factors and batch effects should not be underestimated as they can significantly contribute to the difficulty in replicating scientific findings between cohorts including respiratory medicine [100]. Although computational methodologies and analytical tools are constantly improving with advances in areas such as artificial intelligence and information science, there remain important challenges in accurately separating biological signals from technical noise, particularly for complex diseases such as COPD which are often influenced by multiple factors. Contamination is an important issue with NGS, and care with sample acquisition, handling and processing including the incorporation of sequencing controls at each step are paramount to identify potential contaminants. An additional bioinformatics approach may be added, especially to identify contaminant organisms and exclude them from downstream data analysis [79,101]. It can be difficult to distinguish between protein, gene, and epigenetic perturbations from batch effects, contamination, and technical heterogeneity [102]. Bioinformatic approaches that integrate large amounts of data into models and/or network representations may help address the issue, however, no single approach is suitable for all cases. Many different algorithms are available, including clustering, network analysis, data reduction (PCA), and Bayesian analysis [11,103,104]. The overall lack of standardized biostatistical and algorithmic procedures can itself lead to uncertainty about the validity of results and difficulty in reproducing them, making it important for bioinformatic researchers to assess the sensitivity and optimal network thresholds of their implementations. Ensuring replication and experimental validation of results remains a priority for this field. Most commonly available data integration tools are generally accessible as source code and require computer programming expertise to understand and apply in clinical settings. Such issues may be addressed through the development of iterative software tools with a focus on clinical accessibility and implementation that incorporates preprocessing techniques such as normalization, filtering, and feature selection.

*System representation:* Networks, or graphs, are graphical representations of complex data. In these networks, nodes represent the elements of the system being studied (such as genes, proteins, or individuals), and edges, or links, connect nodes that interact in some way (such as through causation or correlation). These networks are not fixed templates but can be customized to the specific system being studied and are used to make inferences about the dynamic behavior of the system in response to perturbations of critical network elements, for instance, the effect of antibitoics on microbes in the COPD airway. Systems biology, which is typically used to study experimental models of disease may also provide useful insights for clinical medicine. Network medicine can identify disease biomarkers and/or drug targets, which are key nodes whose perturbation can shift the state of the biological system from health to disease or vice versa [105,106].

*Drug discovery challenges*: Network pharmacology is a drug discovery approach that aims to simultaneously target multiple nodes in a disease-specific biological network with small molecules to restore normal, healthy dynamic function. This approach has the potential to improve efficacy and reduce the toxicity of therapeutic intervention, but further methodological development is needed. In one example of multi-omic integration addressing the COPD-bronchiectasis association, *Huang* and colleagues proposed five endoytypes based on combined microbiome/proteome profiles, to speculate on “targetable” treatment approaches [92]. Further, concurrent integration of gut and lung microbiomes allows for the capture of complex interactions between distinct anatomical sites, to stratify patients into “high-“ and “low-“ gut-lung interaction groups in a non-cystic fibrosis bronchiectasis study [107]. Such approaches should be applied to COPD. Importantly, the lack of standardized biostatistical and algorithmic procedures between studies creates uncertainty about the validity of results and hinders reproducibility, making experimental validation a major priority for such analyses.

## 6. Clinical Application and Future Directions

Advances in sequencing technologies have enabled a deeper understanding of the lung microbiome in COPD. The affordability and availability of these technologies, along with the simplification of bioinformatics processes, have made it possible to consider their use in clinical settings. While their use in clinical practice remains at a relatively nascent stage, NGS and multi-omic integration identifies individuals with high-risk COPD and poorer outcomes which may aid in prospective treatment stratification [16,52]. For instance, dysbiosis of the airway microbiome, particularly in COPD with low eosinophils and those receiving fluticasone-based ICS treatment, have the highest risks of pneumonia [73]. Airway microbiomes dominated by neutrophilic-*Haemophilus* profiles are linked to poorer prognosis, while *Prevotella*-dominance relates to better clinical outcomes [15,49].

Therapeutic targeting of the lung microbiome should be considered for clinical benefit; however, important practical challenges need to first be addressed [108]. Manipulating the microbiome with the idea of restoring a “healthy” ecosystem has proven successful in the treatment of gastrointestinal diseases. For example, fecal microbiota transplantation (FMT) has been a very successful treatment for recurrent *Clostridium difficile* infection, and probiotics may prevent antibiotic-associated diarrhea [109,110]. The use of a *Streptococcal* nasal spray to alter the upper airway microbiome has been attempted in children with recurrent otitis media, albeit with mixed results [111,112]. Microbiome-based therapy is a growing field, and integrative multi-omic approaches will assume even greater importance as they provide a more comprehensive characterization of the microbial environment for future precision therapy in COPD. Integrating multi-omic data is highly relevant in COPD, as identifying microbial-metabolite-host signatures has already been shown to provide a holistic approach with clinical relevance [37]. Importantly, however, the high throughput data generated by multi-omics technologies necessitate the early involvement of statisticians and bioinformaticians for optimal, standardized data processing and analysis [98]. Analytical challenges continue to include topological differences in data collection, variation in individual omic data composition (absolute versus relative quantification), and methods utilized for data integration. The disparity in statistical methods employed and the types of datasets used for integration further heighten the complexity of data interpretation [10,97,98]. Moreover, it remains difficult to infer causation despite having large datasets, especially if collected at a single time point. Overcoming these challenges requires better validation, including experimental, which will ultimately allow for better utilization of multi-omics data and an improved understanding of the mechanisms underlying the onset and progression of COPD.

**Figure 2:**
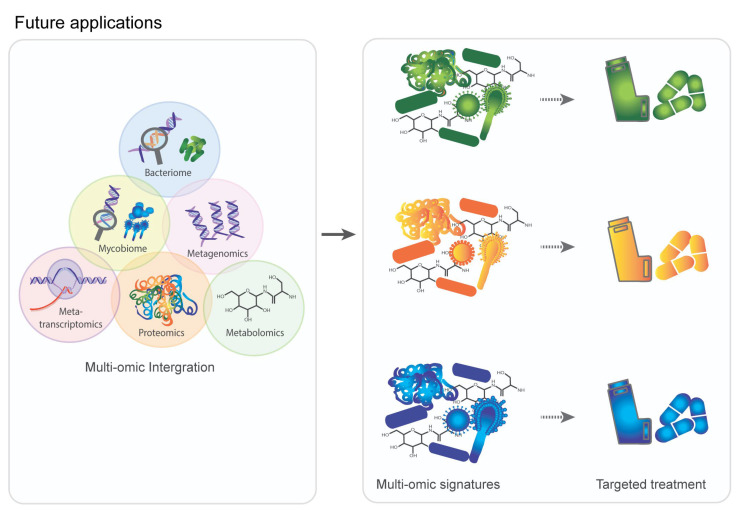
Future applications of integrative multi-omic approaches in COPD will lead to individualized multi-omic signatures with the potential for personalized COPD treatment.

The implementation of systems medicine, which involves the integration of various “omics” data and the use of interdisciplinary teams, requires significant change to healthcare systems. These changes include the creation of specialized data storage facilities, the development of standard analytical pipelines, and the training of new staff with expertise in NGS, multi-omics, and computational biology. The Helper Context-aware Engine System (HCES) is an online tool that aims to support patients and doctors in managing chronic diseases at all levels of risk [113]. It uses a Bayesian network algorithm to depict the dependencies between COPD symptoms (attributes) in order to address the limitations of the naive Bayesian hypothesis, which assumes independence between attributes. Using similar tools in the future, integrating multi-omics can improve the prediction of risk factors and provide computer-aided support applications for disease monitoring in COPD. Critically, however, such change comes with significant costs, particularly in developing countries where the COPD burden remains high [114]. There is a clear need to embrace a holistic scientific approach (as opposed to a traditional reductionist research strategy) that leverages each distinct “-omic” dataset derived from multi-omic studies in COPD. By integrating information from multiple biological levels (such as genes, molecules, cells, organs, and the environment) and using multi-omics for assessment into a single mathematical model, we may better understand the chain of events leading to observable phenotypic manifestations in COPD and uncover novel methods of intervention for the prevention and/or treatment of this disease (Figure 2).

## 7. Conclusions

Studies to date in COPD demonstrate a significant relationship between the microbiome and its host (Figure 1). An increased abundance of *Prevotella* is associated with lower airway symptoms and better lung function, while *Haemophilus* relates to exacerbations and mortality even in stable COPD. Metabolomic studies reveal distinct airway metabolomic signatures related to mucus hypersecretion, NETS, and dysregulation of sphingolipids in COPD. Changes in the bacteriome and metabolites have been observed with COPD treatment during exacerbations and with ICS and long-term macrolide use in stable COPD (Figure 1). By employing different omics techniques, these works delineate potential pathways that associate with an increased risk of COPD progression. In order to gain a more comprehensive understanding of the role of microbes in COPD, future studies must move beyond single kingdom approaches and instead employ multi-kingdom and inter-organ views using NGS and multi-omics in longitudinal and interventional cohort studies, including clinical trials. This will aid the rapid development of endophenotyping, biomarkers, and therapeutics with the potential of realizing precision medicine in COPD.

## Data Availability

Not applicable.
